# Higher levels of TIMP-1 expression are associated with a poor prognosis in triple-negative breast cancer

**DOI:** 10.1186/s12943-016-0515-5

**Published:** 2016-04-30

**Authors:** Guangcun Cheng, Xuemei Fan, Mingang Hao, Jinglong Wang, Xiaoming Zhou, Xueqing Sun

**Affiliations:** Department of Ophthalmology, Shanghai Ninth People’s Hospital, Shanghai Jiao Tong University School of Medicine, Shanghai, 200011 China; Department of Biochemistry and Molecular Cell Biology, Shanghai key Laboratory of Tumor Microenvironment and Inflammation, Hongqiao International Institute of Medicine, Shanghai Jiao Tong University School of Medicine, Shanghai, 200025 China

**Keywords:** Triple-negative breast cancer, TIMP-1, Poor prognosis, G1 phase

## Abstract

**Background:**

Tissue inhibitor of metalloproteinases-1 (TIMP-1) is a multifunctional protein that can directly regulate apoptosis and metastasis. In this study, we investigated the functional and molecular mechanisms by which TIMP-1 influences triple-negative breast cancer (TNBC).

**Methods:**

The expression level of TIMP-1 in breast cancer tissues was analyzed using the ONCOMINE microarray database. The overall survival of patients with distinct molecular subtypes of breast cancer stratified by TIMP-1 expression levels was evaluated using Kaplan–Meier analysis. Bisulfate sequencing PCR (BSP) was used to analyze the methylation status of the TIMP-1 promoter. Real-time-PCR (RT-PCR), Western blot and ELISA assays were used to evaluate gene and protein expression in cell lines and human tissue specimens. In addition, TIMP-1 function was analyzed using a series of in vitro and in vivo assays with cells in which TIMP-1 was inhibited using RNAi or neutralizing antibodies.

**Results:**

We found that serum TIMP-1 levels were strongly enhanced in patients with TNBC and that elevated TIMP-1 levels were associated with a poor prognosis in TNBC. However, TIMP-1 levels were not significantly associated with overall survival in other subtypes of breast cancer or in the overall population of breast cancer patients. We also report the first evidence that the TIMP-1 promoter is hypomethylated in TNBC cell lines compared with non-TNBC cell lines, suggesting that aberrant TIMP-1 expression in TNBC results from reduced DNA methylation. RNAi-mediated silencing of TIMP-1 in TNBC cells induced cell cycle arrest at the G1 phase and reduced cyclin D1 expression. In addition, mechanistic analyses revealed that the p-Akt and p-NF-κB signaling pathways, but not the GSK-3β and MAPK1/2 pathways, are associated with TIMP-1 overexpression in TNBC cells. Moreover, neutralizing antibodies against TIMP-1 significantly decreased the rate of tumor growth in vivo.

**Conclusions:**

Our findings suggest that TIMP-1 is a biomarker indicative of a poor prognosis in TNBC patients and that targeting TIMP-1 may provide an attractive therapeutic intervention specifically for triple-negative breast cancer patients.

## Background

Human breast cancer is a heterogeneous disease, and predicting treatment response and clinical outcomes is typically based on specific clinical and pathological features [1]. Breast cancer is molecularly classified into the luminal-A, luminal-B, HER2-overexpressing (HER2+) or triple-negative subtypes. Triple-negative breast cancer (TNBC) refers to a subtype of breast carcinoma characterized by the lack of expression of the 3 receptors most commonly targeted by standard breast cancer therapy: estrogen receptor alpha (ERα), progesterone receptor (PR) and human epidermal growth factor receptor 2 (HER-2) [2]. In practice, TNBC is often used as a surrogate name for basal-like breast cancer [3]. There is currently no consensus on the optimal immunohistochemistry (IHC) panel to use to characterize basal-like tumors [4]. Although systematic therapeutic approaches have reduced cancer-specific mortality, TNBC is associated with relatively poor clinical outcomes compared with other subtypes of breast cancer [5, 6]. In recent years, there has been a focus on further characterizing the various molecular markers and biomarkers associated with TNBC, including EGFR, VEGFR, c-Myc, C-kit, Poly (ADP-ribose) polymerase-1, HSP90, TOP-2A and spleen tyrosine kinase (SYK) [7, 8]. These biomarkers might be valuable prognostic indicators and might represent potential therapeutic targets of TNBC treatment. Identifying novel biomarkers of TNBC might further contribute to the development of effective TNBC treatment approaches.

Tissue inhibitor of metalloproteinases-1 (TIMP-1), a member of the TIMP family of proteins comprising TIMP-1, 2, 3 and 4, was identified 2 decades ago and was initially characterized as an endogenous inhibitor of matrix metalloproteinases (MMPs) [9–12]. TIMP-1 has long been recognized for its role in extracellular matrix remodeling [13]. Emerging evidence indicates that TIMP-1 is frequently overexpressed in several types of human cancers, including prostate cancer [14], lung cancer [15], melanoma [16], glioblastoma [17] and breast cancer [18, 19]. As a cytokine and a key regulator of ECM degradation, TIMP-1 has multiple functions associated with the tumor microenvironment and cancer progression [20]. In addition to its inhibitory activity against MMPs, TIMP-1 promotes cell proliferation in various cell types [21], including breast cancer cells [22, 23], and it might also be associated with anti-apoptotic activity in breast cancer [24–26]. Although the anti-apoptotic activity of TIMP-1 in other cancers has been well demonstrated, some studies evaluating the role of TIMP-1 in breast cancer cell growth have reported conflicting results [23, 27]. For example, in MDA-435 breast cancer cells, TIMP-1 was reported to promote cell growth by inhibiting MMPs [23]. In contrast, TIMP-1 was reported to inhibit cell growth in MCF-10A normal breast epithelial cells by decreasing cyclin D1 levels [27]. In TIMP-1-deficient mice, mammary epithelial cell proliferation is upregulated [28]. Thus, although several distinct signaling pathways and putative receptors have been implicated in TIMP-1 function [29–32], the mechanisms underlying the role of TIMP-1 in distinct subtypes of breast cancer remain unclear.

To gain new insights into the role of TIMP-1 during breast cancer progression, we examined TIMP-1 expression levels in serum derived from breast cancer patients and evaluated the prognostic value of TIMP-1 using a large publically available clinical microarray database of breast cancer specimens. Interestingly, we observed higher levels of TIMP-1 expression in patients with TNBC compared with control individuals, and this phenomenon was associated with a poor prognosis in TNBC patients. However, TIMP-1 expression levels were not associated with survival in other subtypes of breast cancer or in the overall population of breast cancer patients evaluated. Mechanistic analyses indicated that shRNA-mediated knockdown of TIMP-1 in TNBC cells induced cell cycle arrest at the G1 phase and decreased cyclin D1 levels. Moreover, inhibiting TIMP-1 function prevented tumor growth in mice, suggesting that TIMP-1 inhibition might be a promising therapeutic strategy for treating TNBC.

## Methods

### ONCOMINE microarray datasets

Microarray datasets of invasive breast carcinoma (The Cancer Genome Atlas: Invasive Breast Carcinoma Gene Expression Data, 2011, http://tcga-data.nci.nih.gov/tcga/) and ductal breast carcinoma (The Cancer Genome Atlas: Invasive Breast Carcinoma Gene Expression Data, 2003, http://www.ncbi.nlm.nih.gov/geo/query/acc.cgi?acc=GSE4382) were accessed via the ONCOMINE Cancer Profiling Database (version 4.4.4.4, www.oncomine.org) and were used to investigate TIMP-1 expression in various types of breast cancer.

### Cell culture

The human breast cancer cell lines corresponding to the luminal subtype (MCF-7 and BT474 cells), HER2+ subtype (SK-BR-3) and TNBC subtype (MDA-MB-231, MDA-MB-468, MDA-MB-435 and BT549 cells) were obtained from the American Type Culture Collection (ATCC) and cultured according to the manufacturer’s online instructions. The immortalized epithelial cell line MCF-10A (ATCC) was maintained in DMEM/F12 medium (Invitrogen) supplemented with 5 % horse serum, EGF (20 ng/ml), hydrocortisone (0.5 mg/ml), cholera toxin (100 ng/ml), insulin (10 μg/ml) and 1 % penicillin/streptomycin.

### Real-time PCR

Total RNA was extracted using Trizol reagent (Cat. #15596-026, Invitrogen) and reverse transcribed using the transcriptase cDNA synthesis kit (Cat. #K1662, Fermentas) according to the manufacturer’s instructions. Real-time PCR analysis was conducted using SYBR Premix Ex Taq™ (Cat. #RR420A, TaKaRa, China) and the Applied Biosystems 7500 Fast Real-Time PCR System (ABI, USA). The results were normalized to the GAPDH internal control. The following primers were used: TIMP-1-F: TTGTGGGACCTGTGGAAGTA, TIMP-1-R: CTGTTGTTGCTGTGGCTGAT, GAPDH-F: ACGGATTTGGTCGTATTGGG, and GAPDH-R: CGCTCCTGGAAGATGGTGAT.

### TIMP-1 shRNA lentiviral vectors

TIMP-1 shRNA lentiviral vectors were created by inserting the TIMP-1 target sequences into the GV248 lentiviral vector (GeneChem Company, Shanghai, China). The following TIMP-1 target sequences were used: shTIMP1-1#: ACAGTGTTTCCCTGTTTAT, shTIMP1-2#: AGCGTTATGAGATCAAGAT, and shTIMP1-3#: AGTCAACCAGACCACCTTA. The resulting shRNA lentiviral vectors were transfected into 293 T cells and the viral supernatants were collected and filtered 48 h after the transfection. MDA-MB-468 and MDA-MB-231 cells were infected with the viral supernatant and successfully infected cells were selected using puromycin (0.5 μg/mL) (#P8833, Sigma).

### ELISA

TIMP-1 levels in preoperative patient serum samples and cell-conditioned medium were detected using Quantikine Human TIMP-1 ELISA Kits (Cat. #DTM100, R&D Systems). ELISA assays were conducted according to the manufacturer’s instructions. All the samples were analyzed in 3 wells in each experiment, and each experiment was repeated 3 times. The serum samples were collected from 81 patients prior to surgery. The use of the patient specimens was approved by the Institutional Ethics Committee of Shanghai Ninth People’s Hospital affiliated with Shanghai JiaoTong University School of Medicine, and written consent was obtained from all participants.

### Overall survival (OS) analysis

The Sorlie classification method used in the data set was used to assign patients to the different groups according to clinical breast cancer subtype. OS stratified by expression levels of the gene of interest was evaluated using Kaplan–Meier analysis, and comparisons between groups were evaluated using log-rank tests. The statistical analysis was performed according to the manufacturer’s instructions [33] (http://kmplot.com/analysis/).

### DNA isolation, bisulfite conversion and methylation analysis

DNA was extracted using the Beyotime^®^ Genomic DNA Mini Preparation Kit (Cat. #D0063, Beyotime, China). The DNA samples (500 μg) were treated with bisulfite using the EZ DNA Methylation Gold™ Kit (Cat. #D5006, ZymoResearch, USA). Bisulfite-converted genomic DNA was amplified using ZymoTaq™ DNA polymerase (Cat. #E2001, CA, USA). The BSP specific primers were designed according to the location of the TIMP-1 CpG islands. The primer sequences are as follows: TIMP-1-BSP-F: TGTATAATAAATGTTGAAGGGTTGAATTA and TIMP-1-BSP-R: ACCATCAATACAAAAACCAAAAAAC. The PCR products were inserted into the pCR2.1 vector using the TA cloning Kit (Cat. #K2020-20, Invitrogen, USA), and 10 clones were sequenced by MeiJi Company (Shanghai, China).

### Cell cycle analysis

Cells cultured in 6-well plates were harvested, washed once in PBS and fixed in 70 % ethanol for 48 h at 4 °C. The nuclei were stained with 50 μg/ml propidium iodide (PI) in 1 % Triton-X100/PBS containing 100 μg/ml DNase-free RNase, and the DNA content was analyzed using flow cytometry with the FACSCalibur platform (Becton Dickinson, San Jose, USA). The proportion of cells in each phase of the cell cycle was determined using the ModFit LT program (Verity Software House, USA).

### Colony formation assay

For the colony formation assays, 1 × 10^3^ cells were plated into 6-well plates and cultured for 10 days. At the end of the culture period, the cells were fixed with methanol for 30 min and stained with crystal violet for 30 min. The plates were washed several times with water, and the images of the optical density of the cells were captured using a digital camera.

### Invasion assay

Cell invasion was examined using a reconstituted extracellular matrix membrane (Cat. #354480, BD Biosciences, San Jose, CA). Cells suspended in serum-free media at a concentration of 3 × 10^4^ cells/0.5 ml were placed in the upper chambers, and complete media containing 10 % fetal bovine serum (FBS) and 1 % antibiotics (Invitrogen Corp., Carlsbad, CA) was added to the lower chambers. The chambers were incubated for 18–24 h at 37 °C and 5 % CO_2_. After the incubation, the medium was completely removed from the upper and lower chambers, and the purple residue indicative of noninvasive cells was gently removed from the upper chamber using a cotton-tipped swab. Next, the chambers were fixed with methanol for 30 min and stained with crystal violet for an additional 30 min. The cells were counted in images of the membrane that had been captured using a microscope (Zeiss) with a 10x objective lens.

### Western blot

The Western blot assays were conducted as previously described [34]. Briefly, cells were washed with cold PBS 3 times and harvested in RIPA buffer [1× PBS, 1 % NP40, 0.5 % sodium deoxycholate, 0.1 % SDS, phosphatase inhibitor cocktail (Roche, Indianapolis, IN), 0.1 mg/ml PMSF and 1 mM sodium orthovanadate]. Proteins extracted from the cells or tissue lysates were resolved using 8 %, 10 % or 12 % SDS-polyacrylamide gel electrophoresis, transferred to a nitrocellulose membrane, blocked in 5 % nonfat milk and blotted with the appropriate antibody.

### Xenograft models

The mouse xenograft tumor assays were performed in the animal center of Shanghai Jiao Tong University School of Medicine after obtaining approval from the Shanghai Medical Experimental Animal Care Commission. Twenty 6-week old female mice were obtained from the Shanghai Medical Experimental Animal Care Commission. All the animal experiments were performed in a designated animal center. The mice were subcutaneously injected (2 injection sites per mouse) with 1 × 10^6^ MDA-MB-468 cells and divided into 2 groups (*N* = 10 for each group). Five days later, the mice were injected with a neutralizing antibody against TIMP-1 (10 μg per 25 g of body weight) (Cat. #AF970, R&D Systems) or the IgG control, and the injections were repeated once a week for 4 weeks. Tumor volumes were measured regularly using the formula V = 0.5 × L × W^2^, where L was the longest diameter, and W was the shortest diameter, before the animals were sacrificed, and the tumors were isolated.

## Results

### TIMP-1 expression was significantly elevated in breast cancer

**Fig. 1:**
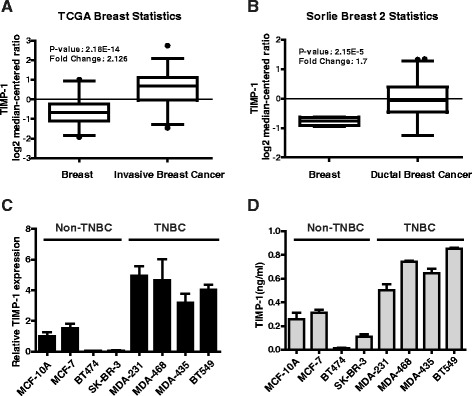
TIMP-1 is highly expressed in breast cancer and is expressed at particularly high levels in TNBC. **a** TIMP-1 gene-centric expression analysis using Oncomine. TIMP-1 is significantly overexpressed in invasive breast cancer tissue compared with normal breast tissue in samples from the TCGA Breast cancer microarray database (*p* = 2.18E-14). **b** Similar results were observed in the Sorlie Breast 2 microarray data (*p* = 2.15E-5). **c** The expression level of TIMP-1 mRNA in various breast cancer cell lines was evaluated using Real-time PCR. The data are presented as the mean ± SD. **d** TIMP-1 protein levels in cell culture medium were detected using ELISA assays. The results demonstrated that TNBC cells express higher levels of TIMP-1 compared with non-TNBC cells. The data are presented as the mean ± SD

To characterize the role of TIMP-1 in breast cancer, we analyzed TIMP-1 mRNA expression in breast cancer specimens from the publicly available cancer microarray database ONCOMINE (https://www.oncomine.org/). We found that TIMP-1 expression was significantly increased in invasive breast carcinoma (Fig. 1a) and ductal breast carcinoma (Fig. 1b) compared with normal breast tissues. We also evaluated the levels of TIMP-1 mRNA and protein in breast cancer cell lines and found that TIMP-1 expression was significantly elevated in the TNBC cell lines (MDA-MB-231, MDA-MB-468, MDA-MB-435 and BT549) compared with the luminal (MCF-7 and BT474) and HER2+ breast cancer cell lines (SK-BR-3) and the normal epithelial cell line (MCF-10A) at the mRNA and protein levels (Fig. 1c and d).

TIMP-1 was initially identified in human serum derived from skin fibroblasts in 1975 [35]. Based on this finding, we assessed the association between serum levels of TIMP-1 breast cancer clinical parameters, including age (< or ≥50 years), T status, lymph node metastasis, and the ER, PR and HER2 status (Table 1). Serum TIMP-1 were significantly elevated in malignant tissues compared with benign tissues (*p* < 0.001) and in ER-negative breast cancer patients compared with ER-positive patients (*p* = 0.002). Stratifying TIMP-1 levels according to molecular subtype revealed that serum levels of TIMP-1 were significantly higher in patients with the luminal-A (*p* < 0.001), HER2+ (*p* < 0.001) and TNBC (*p* < 0.001) subtypes compared with patients with benign disease. However, there were no significant differences in TIMP-1 levels among the 3 malignant subtypes (*p* > 0.05, data not shown).

**Table 1:** Relationship between clinicopathological features and serum levels of TIMP-1 in breast cancer patients

			TIMP-1 level (ng/ml)			*p* value
		N	Mean	SD	Rang	
All study subjects		81	211.2317	82.4088	40.1078–414.3112	
Diagnostic category						<0.001
	Benign	22	159.8383	58.3779	40.1078–239.5306	
	Malignant	59	230.3953	82.2059	73.2381–414.3112	
Age						0.985
	<50	22	230.1297	79.5451	78.9473–378.5845	
	≧50	37	230.5533	84.8323	73.2381–414.3113	
T status						0.386
	≦2	24	215.8122	86.4862	73.2381–414.3113	
	>2	23	238.4916	90.9452	86.1807–404.1802	
Lymph node						0.798
	0	30	221.8737	89.6307	73.2381–414.3113	
	≧1	19	228.2739	76.2754	86.1807–324.1554	
ER						0.002
	Negative	24	268.9795	82.9122	73.2381–324.1554	
	Positive	35	203.9396	71.4596	86.1807–414.3113	
PR						0.030
	Negative	34	250.1643	83.3048	73.2381–324.1554	
	Positive	25	203.5096	74.0452	75.7956–414.3113	
HER2+						0.120
	Negative	38	242.7811	70.3116	86.1807–404.1802	
	Positive	21	207.971	98.1427	73.2381–414.3113	
Ki-67(%)						0.597
	<15 %	20	238.4001	62.2747	142.3562–378.5845	
	≧15 %	39	226.2903	91.2385	73.2381–414.3113	
Molecular subtype						
	Luminal-A	25	230.8728	58.3779	134.5391–324.1554	<0.001
	Luminal-B	10	136.6069	61.2456	73.2381–250.8275	0.327
	HER2+	11	272.8475	78.5198	126.9324–414.3113	<0.001
	TNBC	13	280.6168	74.7404	143.2887–404.1802	<0.001

Serum was collected from 81 patients before surgery, including 22 benign and 59 malignant. Serum TIMP-1 level was detected by ELISA. Before detection, the serum samples were diluted 1:50 with 1 % BSA. ELISA was done according to the manufacturer’s instructions. Student’s T test was used to assess whether the mean of different group has statistically difference

These results suggested that elevated TIMP-1 expression might play an important role in breast cancer development.

### TIMP-1 predicts poor clinical outcomes in patients with TNBC

**Fig. 2:**
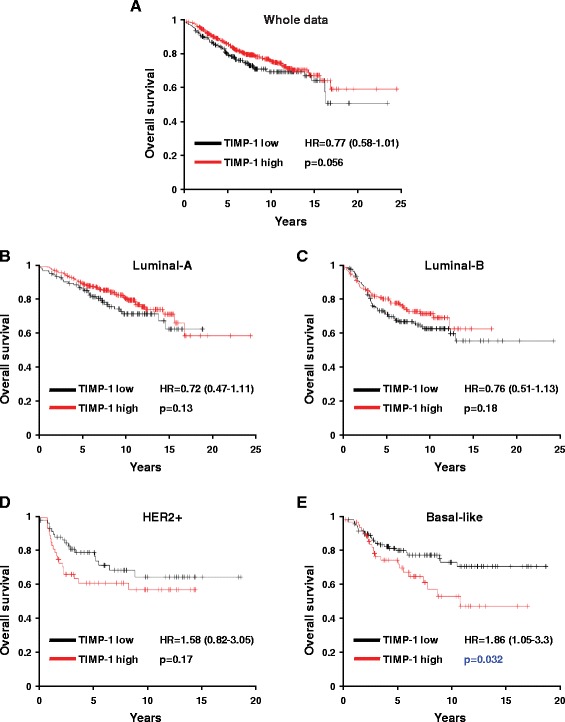
Kaplan-Meier plots of overall survival stratified by TIMP-1 expression levels. **a** Kaplan-Meier plots of overall survival in breast cancer patients from the whole data sets stratified by TIMP-1 expression levels. Data were obtained from the Kaplan-Meier plotter database [33]. The p value was calculated using the log rank test. **b**-**e** Kaplan-Meier plots of overall survival of Luminal-A, Luminal-B, HER2+ and basal-like breast cancer patients. TIMP-1 expression was associated with a poor prognosis in patients with basal-like cancer but not in the overall population or in the other breast cancer subtypes

To further explore the relationship between TIMP-1 and clinical prognosis in patients with breast cancer, we evaluated the prognostic value of TIMP-1 in a large publically available clinical breast cancer microarray database [33] that includes data from 1027 patients (459 luminal-A, 308 luminal-B, 75 HER2+ and 185 TNBC). We found that higher levels of TIMP-1 expression were associated with poor overall survival (OS) in TNBC patients (*p* = 0.032, Fig. 2e) but not in the overall breast cancer population or in the other subtypes evaluated (*p* > 0.05, Fig. 2a-d).

### Upregulation of TIMP-1 in TNBC is associated with promoter hypomethylation

**Fig. 3:**
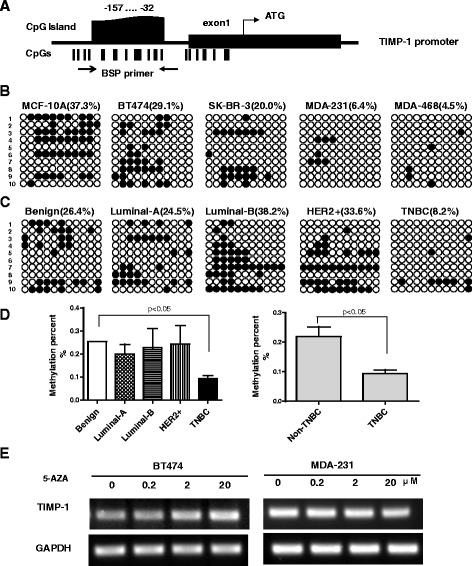
Analysis of TIMP-1 promoter methylation in breast cancer cell lines and tissues. **a** Schematic of the TIMP-1 promoter. The black box indicates exon1 and ‘ATG’ indicates the translational start site. Each vertical bar represents a CpG dinucleotide. The CpG island located between −157 to −32 bp includes 11 CpG sites. The arrows indicate the location of the BSP primers. **b** Methylation status of the TIMP-1 promoter in breast cancer cell lines. BSP analysis was conducted in 10 clones from each cell line. A solid circle represents a methylated CpG site, and an empty circle represents a non-methylated CpG site. The TNBC cell lines (MDA-231 and MDA-468) exhibited lower methylation levels compared with the non-TNBC cell lines (MCF-10A, BT474 and SK-BR-3). **c** Representative images of the methylation status of TIMP-1 promoter in normal tissue and in various subtypes of breast cancer tissues. **d** Quantification of the mean methylation levels observed in the cancer tissues. Methylation was reduced in TNBC tissues compared with the benign, luminal-A, luminal-B and HER2+ tissues (left), collectively referred to as the non-TNBC group (right). The data are presented as the mean ± SD, *p* < 0.05. **e** Expression of TIMP-1 was analyzed using RT-PCR in breast cancer cells treated with various concentrations (0–20 μM) of 5-Aza for 48 h. TIMP-1 expression was restored in BT474 cells treated with 2–20 μM of 5-Aza. In contrast, TIMP-1 expression was not restored in MDA-231 cells treated with 5-Aza due to hypomethylation of the TIMP-1 promoter

DNA methylation is a key epigenetic modification in the mammalian genome that regulates gene expression. To determine if DNA methylation is associated with the transcriptional silencing of TIMP-1 in different subtypes of breast cancer, we analyzed the promoter sequence of TIMP-1. We identified 1 CpG island located between bp −157 and −32 (Fig. 3a) and analyzed its methylation status in breast cancer cell lines and in clinical samples from breast cancer patients. As shown in Fig. 3b and c, the methylation status of the CpG island in the TIMP-1 promoter was greater than 20 % in non-TNBC cell lines (37.3 % in MCF-10A, 29.1 % in BT474 and 20 % in SK-BR-3 cells) and non-TNBC patients (26.4 % in benign, 24.5 % in luminal-A, 38.2 % in luminal-B and 33.6 % in HER2+ patients). In contrast, CpG methylation in the TIMP-1 promoter was less than 10 % in TNBC cell lines (MDA-231 and MDA-468) and TNBC patients. Among the breast cancer patients evaluated in these studies, 5 had luminal-A disease, 3 had luminal-B disease, 3 had HER2+ disease and 3 patients had TNBC. The average methylation status associated with each subtype is presented in Fig. 3c, and the data are summarized in Fig. 3d. These data indicate that methylation of the TIMP-1 promoter is significantly greater in TNBC (*p* < 0.05). In addition, RT-PCR analysis of MDA-231 and BT474 cells treated with various concentrations of 5-Aza-2′-deoxycytidine (5-Aza) for 48 h demonstrated that TIMP-1 mRNA levels increased in BT474 cells but not in MDA-231 cells (Fig. 3e). Together, these findings indicate that high levels of TIMP-1 expression in TNBC might be associated with TIMP-1 promoter hypomethylation.

### TIMP-1 silencing induces cell cycle arrest in the G1 phase in TNBC cells

Based on the observation that TIMP-1 is highly expressed in TNBC cells, we evaluated the role of TIMP-1 in TNBC cells by transfecting MDA-MB-468 and MDA-MB-231 cells with a vector expressing a short hairpin RNA (shRNA) targeting TIMP-1. Three shRNAs, referred to as shTIMP1-1#, −2#, and −3#, were used in these experiments. The shRNA knockdown efficiency of TIMP-1 expression was confirmed using real-time PCR and ELISA assays in both cell lines.

**Fig. 4:**
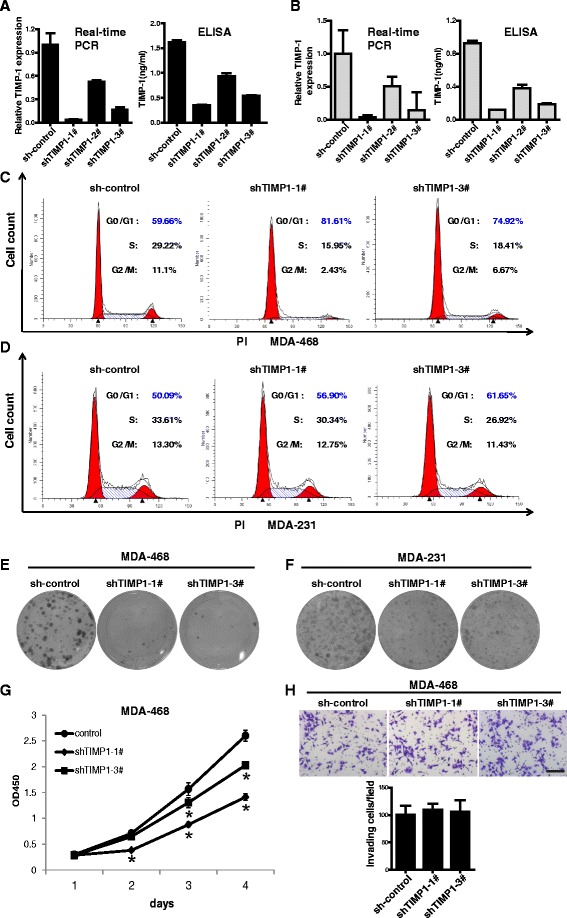
Functional analysis of TNBC cells after TIMP-1 knockdown. **a** Inactivation of endogenous TIMP-1 in MDA-468 cells using shRNA. The knockdown efficiency was confirmed using real-time PCR and ELISA assays. The data are presented as the mean ± SD. **b** Inactivation of endogenous TIMP-1 in MDA-231 cells using shRNAs. shTIMP1-1# and shTIMP1-3# were more efficient in knocking down TIMP-1 compared with the sh-control in both cell lines. The data are presented as the mean ± SD. **c**, **d** Knockdown TIMP-1 expression induced cell cycle arrest at the G1 phase in MDA-468 and MDA-231 cells. **e**, **f** Colony formation was reduced in TIMP-1 knockdown MDA-468 and MDA-231 cells. **g** CCK-8 assays showed that cell growth was restrained in TIMP-1 knockdown MDA-468 cells. * indicates significant difference as compared to control cells (*p* < 0.05). **h** TIMP-1 knockdown did not significantly disrupt cell invasion in MDA-468 cells. Representative images of invasive cells are shown. The data are presented as the mean ± SD; scale bar, 200 μm

To determine the role of TIMP-1 in TNBC cell proliferation, we examined cell cycle distribution using flow cytometry. TIMP-1 knockdown increased the proportion of cells in the G1 phase (81.61 % of shTIMP1-1# cells and 74.92 % of shTIMP1-3# cells vs. 59.66 % of control cells, Fig. 4c), and decreased the proportion of cells in the G2 and M phases compared with the control (Fig. 4c). Similar results were observed in MDA-231 cells (Fig. 4d). In addition, TIMP-1 knockdown significantly reduced colony formation in MDA-MB-468 and MDA-MB-231 cells compared with the control (Fig. 4e and f). CCK-8 assays showed that cell growth was restrained in TIMP-1 knockdown MDA-468 cells (Fig. 4g). However, no differences in cell invasion were observed between TIMP-1 knockdown TNBC cells and control TNBC cells (Fig. 4h).

Together, these results demonstrate that the loss of TIMP-1 expression can induce cell cycle arrest in the G1 phase and reduce colony formation in TNBC cells.

### The Akt signaling pathway is associated with TIMP-1-regulated cyclin D1 expression in TNBC cells

**Fig. 5:**
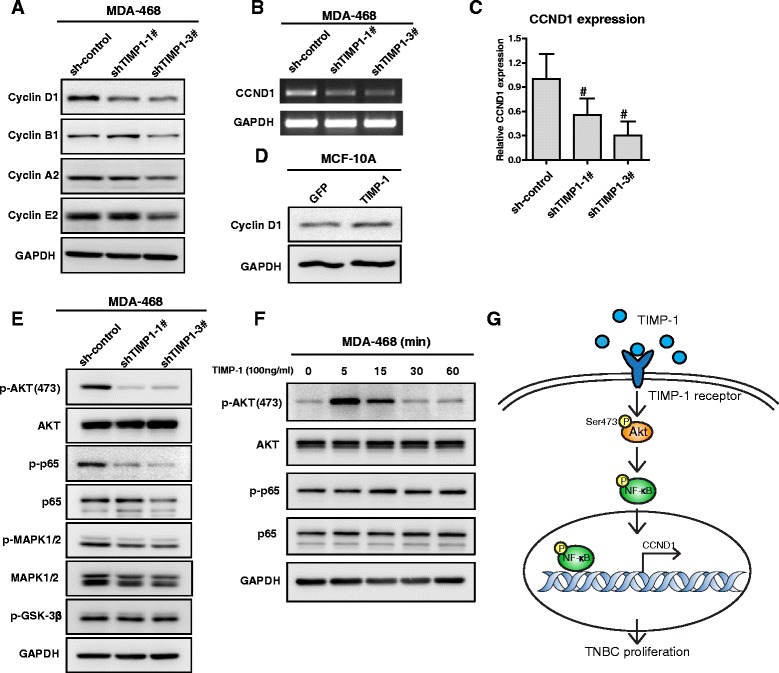
TIMP-1 regulated cyclin D1 expression by activating the Akt signaling pathway in TNBC cells. **a** Western blot analyses of cyclin levels in total cell lysates. Cyclin D1 expression decreased in TIMP-1 knockdown MDA-468 cells, whereas cyclin A2, B1 and E2 levels were not significantly affected. **b**, **c** RT-PCR and real-time PCR experiments were used to evaluate CCND1 mRNA levels in TIMP-1 knockdown MDA-468 cells. The data are presented as the mean ± SD; #, *p* < 0.05. **d** In contrast, cyclin D1 expression was upregulated in MCF-10A cells overexpressing TIMP-1. **e** Akt (60 kDa) and NF-kB (65 kDa) were inactivated in TIMP-1 knockdown MDA-468 cells, but GSK-3β (46 kDa) and MAPK1/2 (42/44 kDa) activation was not significantly affected. **f** Exogenous TIMP-1 (100 ng/mL) activated Akt in MDA-468 cells. **g** Schematic of the potential mechanisms by which TIMP-1 regulates cyclin D1 expression in TNBC cells

To further investigate the molecular mechanism by which TIMP-1 regulates TNBC cell cycle distribution, we examined the levels of cyclin proteins in TIMP-1 knockdown and control MDA-MB-468 cells using Western blot. Cyclin D1, a protein encoded by the CCND1 gene, is required for cell cycle progression from the G1 to the M phase [36]. As shown in Fig. 5a-c, cyclin D1 levels decreased in TIMP-1 knockdown cells. In contrast, TIMP-1 overexpression enhanced cyclin D1 expression in MCF-10A cells (Fig. 5d). The results indicated that TIMP-1 induced cell cycle arrest by upregulating cyclin D1 expression at the mRNA and protein levels.

In TIMP-1 knockdown MDA-MB-468 cells, the Akt (mainly at Ser473 residue) and NF-κB signaling pathways were strongly inhibited, whereas the MAPK1/2 and GSK-3β pathways were unaffected (Fig. 5e). In MDA-468 cells treated with exogenous TIMP-1 (100 ng/mL, Cat#: 970-TM, R&D systems), Akt phosphorylation (primarily at Ser473) increased within 5 min (Fig. 5f), suggesting that the Akt signaling pathway is involved in TIMP-1-induced breast cancer cell proliferation. Figure 5g presents a schematic by which TIMP-1 regulates cyclin D1 expression in TNBC cells via activation of the Akt signaling pathway.

### Blocking TIMP-1 activity with neutralizing antibodies inhibits tumor growth

**Fig. 6:**
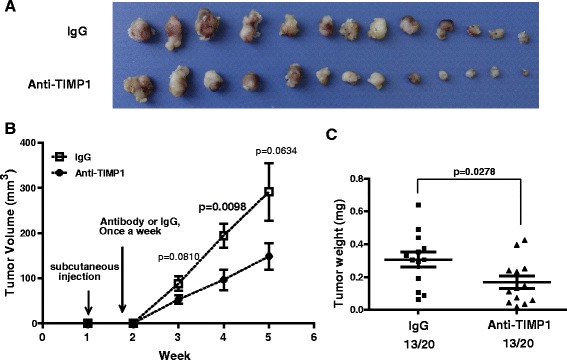
Blocking TIMP-1 activity inhibits TNBC cell growth in vivo. **a** The effect of the neutralizing antibody against TIMP-1 on TNBC cell growth in vivo. 6-week-old female nude mice were subcutaneously injected with 1 × 10^6^ MDA-468 cells. The neutralizing antibody against TIMP-1 or the IgG control was subcutaneously injected into the mice once per week. Representative images of the tumors on week 5 are shown. **b** Tumor volume (mm^3^) was evaluated once per week. Tumor growth was significantly inhibited after 4 weeks in mice injected with a neutralizing antibody against TIMP-1 compared with the control mice (*p* = 0.0098). The data are presented as the mean ± SD of triplicate measurements. **c** Tumor weight was measured at the time the mice were sacrificed. Quantification of total tumor burden demonstrated that tumor growth was suppressed by TIMP-1 inhibition. The data are presented as the mean ± SD; *n* = 13; *p* = 0.0278

To determine if TIMP-1 is involved in tumor growth in vivo, we used a neutralizing antibody to block TIMP-1 activity in TNBC cells. We used this approach rather than engineering TIMP-1 knockdown cells as TIMP-1 is a secreted protein. Ultimately, 13 tumors derived from the cancer cell injections were identified in each group and used for further analysis. A significantly lower rate of tumor growth was observed in mice injected with neutralizing antibodies against TIMP-1 compared with mice injected with the control IgG. The 26 tumors 5 weeks after the tumor cell injections are shown in Fig. 6a. We observed a strong reduction in tumor volume and total tumor burden in mice injected with the neutralizing antibody compared with control mice (Fig. 6b and c). Together, these data suggest that blocking TIMP-1 activity might be an effective approach for treating triple-negative breast cancer.

## Discussion

TIMP-1 is a small secretory glycoprotein with multiple functions, including anti-apoptotic activity and inhibiting matrix metalloproteinases [13, 26]. Numerous studies have demonstrated that TIMP-1 levels are elevated in several types of human cancer, including breast cancer [19]. Breast cancer is a heterogeneous disease composed of distinct molecular subtypes with different phenotypes. Triple-negative breast cancer, which is defined by the absence of ER, PR and HER-2 expression, represents 15 % of breast cancer cases [37]. Among the different subtypes of breast cancers, TNBC is associated with the poorest clinical prognosis, and no effective targeted therapies are currently available [38]. Actually, little is known about the function and molecular mechanism of TIMP-1 in TNBC [39].

In this study, we found that TIMP-1 expression was elevated in TNBC cell lines and TNBC patients compared with non-TNBC cells and non-TNBC breast cancer patients and that increased TIMP-1 expression was associated with a poor prognosis in TNBC patients. Our epigenetic analysis provided the first evidence that elevated TIMP-1 expression in TNBC is associated with a reduction in TIMP-1 promoter methylation. These findings indicate that TIMP-1 expression might be linked to more aggressive subtypes of breast cancer and are consistent with previous studies reporting that TIMP-1 expression is associated with a poor prognosis in breast cancer [40], colorectal cancer [41], laryngeal squamous cell carcinoma [42] and hepatocellular carcinoma [43]. An increase in TIMP-1 mRNA levels induced by 5-Aza treatment has also been observed in melanoma [44] and gestational tissues [45], indicating that promoter methylation mediates the expression of TIMP-1 in various cell types.

As a member of the TIMP family of proteins, TIMP-1 was initially characterized as an endogenous inhibitor of MMPs and A Disintegrin and metalloproteinase domain-containing protein 10 (ADAM10) [46]. However, in recent years, several reports have focused on the cytokine-like functions of TIMP-1 in multiple biological processes [20, 47]. In this study, TIMP-1 down-regulation significantly decreased cyclin D1 expression at both the mRNA and protein levels and disrupted Akt and NF-κB signaling, suggesting that Akt/NF-κB signaling might mediate the effects TIMP-1 exerts on cell cycle regulation in TNBC. Despite previous reports that GSK3β signaling pathway plays a critical role in cyclin D1 degradation [48] and that TIMP-1 activates human breast epithelial cells via the PI3K and MAPK signaling pathways [29], we found that the GSK-3β and MAPK1/2 pathways were unaffected in TIMP-1 knockdown TNBC cells or TNBC cells treated with exogenous TIMP-1. In a recent study, TIMP-1 was reported to phosphorylate Akt at Thr308 in human hematopoietic progenitor cells [47]. Other studies have also reported that TIMP-1 can bind to CD63 or the pro-MMP9/CD44 complex, thereby activating survival pathways in some cells [32, 49]. The physiologic receptor of TIMP-1 remains unclear; therefore, further investigation into TIMP-1 receptors and the intracellular processes mediated by TIMP-1 might provide novel insights into the molecular mechanisms of TIMP-1 in breast cancer cells or other types of cancer cells.

In this study; however, we did not observe defects in cell migration in TIMP-1 knockdown cells. A potential explanation for this finding is that knocking down a single factor is not sufficient to discernably disrupt cell migration in the highly aggressive TNBC cell lines we evaluated.

As the role of TIMP-1 in promoting proliferation in various cell types has been well established, efforts have been put forth to evaluate the effect of blocking TIMP-1 signaling in inflammation-associated diseases by targeting CD63 [31]. As TIMP-1 is secreted in the tumor microenvironment, we used a TIMP-1 neutralizing antibody to block TIMP-1 activity rather than use TIMP-1 knockdown cell lines. We found that the inhibition of TIMP-1 activity markedly suppressed tumor growth in mice, consistent with observations in mouse models of prostate cancer [50]. Targeting TIMP-1 or its receptor is widely used in the treatment of immune disease. In this study we investigated the potential use of TIMP-1 antibody in cancer therapy.

## Conclusions

TIMP-1 was highly expressed in TNBC patients and was associated with a poor prognosis. The TIMP-1 promoter was hypomethylated in TNBC cells, resulting in an increase in proliferation and cyclin D1 levels via the p-Akt and p-NF-κB pathways. Treatment with a neutralizing antibody against TIMP-1 significantly decreased tumor growth in vivo. In summary, our results suggest that TIMP-1 might serve as a prognostic biomarker indicative of poor outcomes and be an effective therapeutic target of TNBC treatment.

## Abbreviations

BSP: Bisulfate sequencing PCR
HER-2: Human epidermal growth factor receptor 2
MMPs: Matrix metalloproteinases
TIMP-1: Tissue inhibitor of metalloproteinases-1
TNBC: Triple-negative breast cancer
